# Nanoparticle-Based Delivery of Tumor Suppressor microRNA for Cancer Therapy

**DOI:** 10.3390/cells9020521

**Published:** 2020-02-24

**Authors:** Clodagh P. O’Neill, Róisín M. Dwyer

**Affiliations:** 1 Discipline of Surgery, Lambe Institute for Translational Research, National University of Ireland Galway, H91 YR71 Galway, Ireland; c.oneill15@nuigalway.ie; 2 CÚRAM, SFI Research Centre for Medical Devices, National University of Ireland Galway, H91 W2TY Galway, Ireland

**Keywords:** tumor suppressor miRNA, cancer therapy, nanoparticle delivery

## Abstract

Improved understanding of microRNA expression and function in cancer has revealed a range of microRNAs that negatively regulate many oncogenic pathways, thus representing potent tumor suppressors. Therapeutic targeting of the expression of these microRNAs to the site of tumors and metastases provides a promising avenue for cancer therapy. To overcome challenges associated with microRNA degradation, transient expression and poor targeting, novel nanoparticles are being developed and employed to shield microRNAs for tumor-targeted delivery. This review focuses on studies describing a variety of both natural and synthetic nanoparticle delivery vehicles that have been engineered for tumor-targeted delivery of tumor suppressor microRNAs in vivo.

## 1. Introduction

Research on microRNAs (miRNAs) has advanced dramatically since the first report of these short non-coding sequences by Ambros et al. [1] in 1993, with over 2000 currently annotated human sequences on MiRbase [2]. MiRNAs are powerful gene regulators believed to impact almost all human physiological pathways in health and disease, from embryogenesis to carcinogenesis. In the cancer setting, miRNAs play a critical role through the negative regulation of tumor suppressors, thus supporting cancer progression (i.e., oncomiRs) or through negative regulation of oncogenes (i.e., tumor suppressor miRNA (TS miRNA), the latter of which will be the focus of this review.

The canonical pathway of miRNA gene regulation is cleavage or repression of a target mRNA via binding with the RNA-induced silencing complex (RISC). MiRNAs most commonly target the 3′UTR of mRNA. Complete complementarity with the target mRNA is not required for binding, and each miRNA can have several mRNA targets [3]. Understanding miRNA-mediated downregulation of tumor progression has resulted in immense interest in the potential for therapeutic application of these sequences. For example, let-7, along with lin-4, were the first discovered miRNAs in *Caenorhabditis elegans* [1]. Let-7 has a powerful tumor-suppressive capacity, including repression of oncogenes such as RAS and high mobility group AT-hook 2 (HMGA2) in humans [3]. More recently, miRNA-34a has also emerged as a potent tumor suppressor. MiR-34a has a synergistic relationship with the tumor-suppressing p53 pathway by negatively regulating epithelial–mesenchymal transition (EMT) [4]. During tumor progression, TS miRNA expression is depleted, allowing oncogenic pathway signalling to progress.

**Figure 1:**
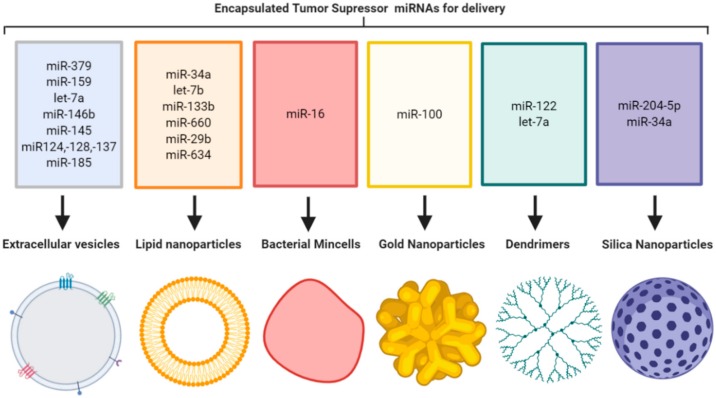
Tumor suppressor miRNA encapsulation in nanoparticle formulations for delivery to primary tumors and metastases (image created using Biorender.com—paid subscription).

Replenishing TS miRNA at the tumor site is an attractive option for the treatment of cancer. Since miRNAs are short single-stranded sequences with unprotected 3′-hydroxy and 5′-phosphate ends that lead to easy degradation by ribonucleases [5,6], they are only transiently expressed and have relatively short half-lives. Combined with a lack of tumor targeting, these are major limitations for cancer therapy. This lack of miRNA stability is overcome by binding to proteins such as argonaute-2 (AGO2) or encapsulation in naturally occurring vesicles such as extracellular vesicles (EVs). Developing on these natural mechanisms, there is a rationale to employ a delivery vehicle that can protect the unstable TS miRNA and deliver it at high levels directly to the tumor site while sparing healthy tissue. Recently nanoparticles have arisen as an attractive option for tumor-targeted delivery of miRNAs. A range of nanoparticle formulations have been employed in an effort to achieve this, including organic-based lipid nanoparticles (LNPs), naturally occurring EVs, genetically created bacterial minicells, and inorganic materials such as silica, gold and polyamidoamine (PAMAM) dendrimers, that are synthetically manufactured to produce nano-sized delivery vehicles (Figure 1).

The ideal nanoparticle delivery vehicle should be safe and well-tolerated, have tumor-targeting capabilities with low off-target effects, and should be easily taken up by cancer cells and deliver high levels of the TS miRNA. Physical properties of the nanoparticle such as size, charge and chemical composition govern these attributes and are discussed in detail throughout this review.

### 1.1. Organic Lipid Nanoparticles

Liposomes are lipid vesicles consisting of one or more phospholipid bilayers encapsulating an aqueous solution. Being amphipathic, liposomes can bind both hydrophobic and hydrophilic molecules, making them attractive drug delivery vehicles that have been utilised in the pharmaceutical industry for many years [7]. Liposomes are the basic unit for all lipid nanoparticles (LNPs). There has been significant research using LNPs to deliver TS miRNA. LNPs can be differentiated based on vesicle charge. Cationic lipids incorporated into LNPs facilitate strong binding to the anionic phosphate backbone of miRNAs and can provide more efficient delivery by binding to anionic molecules on the target cell surface. However, due to this high reactivity with anionic molecules, there have been reports of immunogenicity [8]. Neutral LNPs, as the name suggests, have no charge and are believed to be less immunogenic. LNPs are usually modified with other molecules, including hyaluronic acid (HA) and polyethylene glycol (PEG), to improve characteristics such as tumor targeting and stability [9].

### 1.2. Extracellular Vesicles

Extracellular vesicles (EVs) are naturally occurring nanoparticles released by all cells and play an important role in cell communication by transporting encapsulated proteins, lipids and nucleic acids between cells [10]. The term “extracellular vesicle” encompasses a variety of vesicle types that differ in size and biogenesis pathway, including exosomes (30–120 nm), microvesicles (100–1000 nm) and apoptotic bodies (>1000 nm) [11]. EV cargo is characteristic of the cell of origin. MiRNAs are naturally encapsulated and transported by EVs; consequently, EVs are an emerging exciting contender for use as delivery vehicles to introduce TS miRNA at the tumor site. In this review, EVs from a variety of cell sources are discussed, including mesenchymal stem cells (MSCs), natural-killer (NK) cells, tumor cells and monocytes [12,13,14,15].

### 1.3. Bacterial Minicells

Bacterial minicells are anucleate nanoparticles created by shutting down cell division genes in parental bacterial cells [16,17]. The minicells go through an extensive purification process to remove any bacterial cells. The end product is a nanoparticle, approximately 400 nm in size, that can load therapeutic agents for delivery to a tumor [17]. Minicells do not have natural tumor targeting ability and are cleared by the immune system; however, they can be conjugated to tumor-specific antibodies. Bispecific antibodies (BsAb) consist of two antibodies bound via their fragment crystallizable (Fc) regions. One arm of the BsAb recognizes the O-antigen of lipopolysaccharides (LPS) in the minicell membrane while the other targets a cancer-specific antigen such as epidermal growth factor receptor (EGFR) to support tumor-specific uptake [16].

### 1.4. Inorganic Nanoparticles

Inorganic materials have also been utilised as the building blocks for NPs to deliver TS miRNA, including silicon derivatives and a variety of metals. Silica (SiO_2_) is the oxidised form of the element silicon present in the earth’s crust. Silica sub-units can be arranged to form NPs with varying size pores, ideal for delivery and release of drugs and miRNA. Metal-based NPs are also employed for delivery of TS miRNA. Dendrimers are a class of synthetic branched polymers with uniform nanostructure, allowing the encapsulation of drugs and other molecules. Polyamidoamine (PAMAM) dendrimers are branched repeating inorganic units of amide and amine and were the first commercialised dendrimers [18]. PAMAM dendrimers have been utilised to encapsulate TS miRNA for delivery to the tumor site. Most inorganic based NPs are modified by conjugation with antibodies [19,20], or encapsulated in natural vesicles [15], to produce NPs with clinically relevant properties, including tumor-targeting ability.

This review focuses on a variety of both natural and synthetic nanoparticle (NP) delivery vehicles that have been employed for tumor-targeted delivery of TS miRNA in vivo.

## 2. Tumor Suppressor miRNA Delivery Via Lipid-Based Nanoparticles

Lipid nanoparticles were the first vehicles used to deliver TS miRNA in pre-clinical models. In 2011, Wu et al. [21] delivered cationic lipoplexes containing pre-miR-133b and a fluorescent dye, cyanine-5 (Cy-5), to non-small-cell lung cancer (NSCLC) via intravenous (IV) administration (Table 1). MiR-133b is a TS miRNA downregulated in NSCLC. In normal tissue, it targets myeloid cell leukaemia 1 (MCL1), which is anti-apoptotic and, as a result, lowers cancer cell drug sensitivity [22]. A biodistribution study showed that 30% of fluorescence accumulated in the lungs; however, 50% accumulated in the liver. Ex vivo analysis of the lung tissue indicated there was no inflammation after injection of lipoplexes (H&E staining), and there was a 52-fold increase of miR-133b expression in treated mice compared to control mice. This study did not investigate the therapeutic efficacy of miR-133b delivery but showed the feasibility of TS miRNA encapsulation in a lipid formulation and its accumulation in the lungs in vivo.

Subsequently, the same group employed cationic lipoplexes to deliver miR-29b into the lung tumor environment in a mouse model of the disease [27]. MiR-29b has been shown to be downregulated in many cancers, including lung cancer, with its molecular targets including DNA methyltransferases (DNMT) [38]. After sequential dosing via IV injection of lung tumor-bearing mice with miR-29b-lipoplexes for two weeks, the treated mice had significantly smaller tumors than those injected with a negative control, NC-lipoplexes. Ex vivo analysis of tumor tissue revealed an almost 5-fold increase in miR-29b expression in the treatment group and a significant decrease in three oncogenic targets, CDK6, DNMT3B and MCL1. The group also carried out cytotoxicity analysis of major organs after administration of miR-29b-lipoplexes and found no difference between treatment and control groups [27].

Cationic LNPs were employed to deliver synthetic double-stranded miR-634 mimics (miR-634-LNPs) to pancreatic tumors in vivo [37]. There was a significant reduction in tumor growth in animals treated intravenously with miR-634-LNPs compared to those treated with control miR-LNPs. To explore potential hepatotoxicity of treatment, the weight of the liver and plasma levels of liver injury markers aminotransferase (AST) and alanine aminotransferase (ALT) were measured. No difference between treated and control mice was detected, suggesting miR-634 was well tolerated. However, AST levels were elevated in both groups receiving LNPs compared to healthy control mice, suggesting some toxicity related to the LNPs.

A cationic LNP formulation with a neutral lipid coating was developed to encapsulate therapeutic miR-660 (CLL660) for delivery to lung tumors in a xenograft model [26]. The aim of the neutral lipid coating was to avoid cationic lipid interaction with proteins resulting in an immune response. MiR-660 is a potent tumor suppressor, downregulating MDM2 and allowing the restoration of normal p53 activity [39]. After intraperitoneal (IP) or IV injection of CLL660, there was a 4-fold and 3-fold increase, respectively, in the expression of miR-660 at the tumor site. Based on these results, the efficacy of CLL660 treatment was tested in three different lung patient-derived xenograft (PDX) severely compromised immune deficient (SCID) mice models. Following IP injection twice a week for four or eight weeks, there was a significant decrease in tumor volume in the treated group. Although off-target delivery of CLL660 to other organs, including lung, liver and spleen, was observed, toxicity studies (blood biochemical analyses, IHC staining, organ weight, cytokine analysis) revealed no negative results, suggesting tolerance of this approach [26].

MiR-34a has been studied extensively in vitro and shown to be a potent tumor suppressor in NSCLC [40]. MiR-34a regulates many aspects of cancer biology, including activation of apoptosis via the p53 pathway and downregulation of cell growth and survival factors such as survivin [41]. There have been a number of studies reporting LNP-based delivery of miR-34a (Table 1). The commercially available neutral lipid-based reagent, “MaxSuppressor™ In Vivo RNA-LANCEr II”, was employed to deliver miR-34a via intra tumoral or systemic injection in NSCLC xenograft-bearing NOD/SCID mice [25]. After sacrifice, tumor histology showed large areas of cell debris from both cohorts (IT, IV) injected with the formulated miR-34a as well as increased caspase-3 expression and reduced Ki-67 expression. However, systemic injection of miR-34a resulted in a lower accumulation at the tumor site than direct injection, suggesting degradation of the miRNA, lack of persistence of the delivery vehicle or off-target accumulation. The safety profile of this formulation was tested by IV injection into immunocompetent BALB/c mice. The blood chemistry tested was within the safe range, suggesting no toxicity to major organs. The immune response was also tested by measuring serum levels of a panel of cytokines, with all levels remaining normal.

The tumor suppressor capacity of miR-34a and let-7b individually was also tested in NSCLC using neutral lipid emulsion (NLE) as a therapeutic miRNA delivery vehicle in vivo [24]. An autochthonous model of NSCLC was employed by inoculating LSL-Kras-G12D mice with an adenovirus for 10 weeks, activating oncogenic KRAS^G12D^ and resulting in lung tumor formation. NLE conjugated to let-7b or miR-34a or a negative control was administered via tail vein injection. Let-7b-treated animals and miR-34a-treated animals had significantly lower tumor burden compared to untreated mice. Ex vivo, let-7b-treated mice showed reduced tumor cell proliferation, but apoptosis was not affected. MiR-34a-treated mice had reduced expression of Ki-67 and an increase in TUNEL positive cells, confirming reduced cell proliferation and increased apoptosis.

LNPs were also used to deliver miRNA-34a to secondary lung metastases in a murine model of melanoma [20]. The nanoparticles composed of liposome-polycation-hyaluronic acid (LPH) were modified with a single-chain variable fragment (ScFv) called glycoprotein 4 (GC4) that facilitated the targeting of lung tumor metastases in vivo. When LPH-GC4 was loaded with miR-34a and injected IV into mice with lung metastases, protein expression of survivin was suppressed and apoptosis was activated in the tumor. LPH-mediated co-delivery of miR-34a, and a combination of three small interfering RNAs (siRNAs) targeting c-Myc, MDM2 and VEGF inhibited tumor growth and had a synergistic effect when compared to delivering the therapeutics individually. Toxicity studies of mouse serum revealed neither formulation resulted in any significant induction of proinflammatory cytokines.

## 3. Tumor Suppressive miRNA Delivery Via Extracellular Vesicles (EVs)

Mesenchymal stem cells (MSCs) are multipotent cells that play an important role in tissue repair and regeneration due to an inherent ability to differentiate into multiple tissue types, including bone, fat and cartilage [42]. There is a lot of interest in using MSCs for cancer therapy as they have both tumor homing and immune evasion capabilities. However, since MSCs are principally regenerative cells and secrete abundant growth factors and chemokines, there are concerns that MSCs may be pro-tumorigenic in some scenarios [43]. Since EVs are believed to represent a fingerprint of the secreting cell, EVs released by MSCs (MSC-EVs) could potentially have the same tumor-targeting and immune evasion capabilities [44,45]. Given the natural packaging of miRNAs into EVs, researchers have engineered MSC-EVs to enrich for tumor-suppressing miRNAs, miR-146b, miR-185 or miR-379, followed by either local (topical or intratumoral (IT)) [30,36] or systemic (IV) [12] administration.

MiR-146b is a known tumor suppressor in many brain malignancies, with targets including tumor necrosis factor receptor (TNFR)-associated factor 6 (TRAF6), matrix metalloproteinase-16 (MMP16) and epidermal growth factor receptor (EGFR) [30,46,47]. In many glioma patients, miR-146b activity is lost, leading to increased invasiveness of the tumor. MSC-EVs expressing miR-146 or a control vehicle was administered via an IT injection into 9L gliosarcomas in rats [30]. Ten days later, there was a significant decrease in tumor volume in treated compared to control animals. Although this proof of concept study employed direct IT injection, EVs do hold significant promise for systemic injection due to being capable of crossing the blood–brain barrier, potentially providing an exciting avenue for the treatment of brain malignancy or secondary brain metastases [48].

MiR-185 acts as a tumor suppressor in oral cancer by regulating the AKT pathway and has low expression in oral squamous cell cancer (OSCC) patient samples [49]. Oral potentially malignant disorders (OMPD), is a precursor to OSCC. The efficacy of MSC-EVs enriched with miR-185 was tested in a chemically-induced model of OMPD by topically administering to the OMPD site (left cheek) three times per week [36]. Ex vivo analysis of the buccal mucosa showed treated animals had a reduction in inflammatory cell infiltration, pro-inflammatory cytokines and chemokines, and levels of phosphorylated Akt and nuclear factor kappa-light-chain-enhancer of activated B cells (NF-kB), and increased expression of cleaved caspase -9 and -3, both of which promote apoptosis. MSC-EV-miR-185 treatment stilled inflammation and, as a result, reduced the number of OSCC cases.

While the previous studies employed local administration, IV administration of MSC–EV–miR-379 has been shown to have a therapeutic impact in a murine model of breast cancer. MiR-379 is a tumor suppressor with established targets including the cell cycle mediator cyclin B1, interlukin-11 (IL-11) and cyclooxygenase (COX)-2 [12]. miR-379 has been shown to be downregulated in breast cancer patients compared to healthy controls [50]. The potential of IV administration of MSC–EV–miR-379 was investigated for breast cancer therapy in mice [12]. Bioluminescent imaging revealed a significant reduction in tumor volume in mice that received the MSC–EV–miR-379 therapy compared to control-treated animals. This study shows the exciting potential of MSC-EVs for systemic therapy of metastatic disease, but further investigation of potential immune interactions and factors controlling tumor-specific targeting is required.

While MSC–EVs have an attractive natural homing/immune evasion phenotype, modification of EVs from other cell sources has also been employed to deliver TS miRNAs. The protein A disintegrin and metalloproteinase 15 (A15) has high binding affinity for integrin α_v_β_3,_ supporting the targeting of α_v_β_3_ overexpressing tumors such as triple negative breast cancer (TNBC) [13]. Monocytes exposed to phorbol 12-myristate 13-acetate (PMA) release EVs with high levels of membrane-bound A15. miR-159 has been shown to be reduced in the sera of breast cancer patients and inhibits breast cancer growth via the wnt signalling pathway [51]. To improve miRNA stability and encourage cell internalization, miR-159 was chemically modified through the addition of hydrophobic cholesterol to the 3′ end (Cho–miR-159) [13]. The targeted nanoparticles were loaded with Cho–miR-159 and the chemotherapeutic agent DOX (Co–A15–EV). An initial biodistribution study in mice showed that A15–EVs had high efficiency in targeting the TNBC tumors compared to unmodified EVs, which had off-target distribution in the liver and kidney. Co-delivery of DOX and miR-159 encapsulated in A15–EVs provided the most potent therapeutic response, with a mean survival rate of 53 days versus 27 days for mice treated with saline.

Let-7a is a well-established tumor suppressor reduced in many cancers, including breast cancer [3]. In normal tissue, it works by downregulating the expression of oncogenic targets, including RAS and HMGA2 [52]. EVs from the human embryonic kidney cell line 293 (HEK293) were investigated as delivery vehicles of let-7a to breast tumors in vivo [23]. HEK293 cells were transfected with the GE11 peptide, which has a high binding affinity for the epidermal growth factor receptor (EGFR) protein, which is elevated in many epithelial cancers, including breast cancer. EV-GE11 showed increased binding at breast tumor sites in animals compared to unmodified EVs. Systemic delivery of EV–GE11–let-7a in vivo resulted in suppression of tumor growth. Analysis of let-7a common targets, RAS and HMGA2, revealed no impact of let-7a, suggesting tumor growth suppression occurred through a different pathway.

A group of TS miRNA has been found to be consistently downregulated in glioblastoma multiforme (GBM), miR-124, miR-128 and miR-137, that regulate multiple oncogenic pathways [14]. GBM is an extremely aggressive brain malignancy with a less than 5% survival rate for patients, demonstrating a clear, urgent need for more targeted and potent therapy [53]. An engineered cluster containing the three biologically synergistic miRNAs (Cluster 3) was tested in vivo [14]. Mice inoculated with GBM cells stably expressing Cluster 3 had smaller tumors, prolonged survival and improved response to chemotherapy compared to mice with WT GBM tumors. EVs isolated from GBM cells stably expressing Cluster 3 were directly injected into GBM tumors of mice, resulting in a significant increase in survival compared to controls. This study provides evidence for the potential to use multiple TS miRNAs to increase the potency of administered EVs.

Interestingly, an EV mimetic coined “exosome–mimetic nanoplatforms” (EMNs) was engineered, consisting of a defined composition of phospholipids and cholesterol, with a view to having a similar structure and function to tumor EVs while avoiding the associated safety concerns and production difficulties [28]. EMNs were loaded with therapeutic miR-145 labelled with the fluorescent tag, Cy-5. MiR-145 is a potent tumor-suppressor downregulated in lung cancer and known to inhibit right open reading frame kinase 2 (RIOK2) and NIN1 binding protein (NOB1) [54]. After IP injection of the ENM–miR-145–Cy-5, a fluorescent signal was detected at the lung tumor sites. The off-target fluorescent signal in the liver, kidneys and spleen was lower than the cohort injected with tumor cell-derived EV–miR-145–Cy-5. No signal was detected in the heart for both systems, suggesting low potential for cardiotoxicity.

## 4. Inorganic-Based Nanoparticle Formulation

Polyamidoamine (PAMAM) dendrimers of repetitively branched amide and amine units show great versatility as the building blocks of miRNA delivery vehicles. PAMAM dendrimer-based nanoparticles were used to deliver let-7a to neuroblastoma tumors in a NOD/SCID murine model [15]. The NP formulation consisted of PAMAM dendrimer (NN) linked to let-7a and labelled with Cy-5 to allow fluorescent imaging. Interestingly the NPs were encapsulated by EVs derived from natural killer (NK) cells. The rationale of using NK–EVs was that they could provide more targeted delivery efficiency due to the presence of the CXCR4 receptor on NK–EV, which encourages migration to target cells, including those in the brain. Bioluminescent imaging showed that mice treated with the NP cocktail had suppression of tumor growth [15].

PAMAM dendrimers were also used to deliver TS miRNA-122 to a mouse model of liver cancer [34]. The nanoparticle system was coined “biodegradable oncosensitive megamer-based (BOMB)”, and consisted of a megamer core made of up of PAMAM dendrimers connected by disulfide bonds. The megamer core was conjugated to PEG using a pH-labile linker (Dlinkm) and was cationic for encapsulation of anionic miRNA. Interestingly, this nanoparticle used environmental stimuli rather than molecular targets for directed delivery of miR-122 to the tumor site. In short, the nanoparticle was stable at the neutral pH in circulation; however, the weak acidic pH of the tumor microenvironment broke the links between the megamer core and PEG, and reduction reactions further stimulated the breakdown of the disulfide bonds of the megamer, releasing miR-122 at the tumor site. The whole BOMB nanoparticle system was then fully biodegraded. The feasibility of the BOMB nanoparticle system encapsulating miR122 was tested by systemic injection in a murine model of liver cancer. Tumor miR-122 expression was 2-fold higher and the tumor volumes were smaller in BOMB-miR-122 treated mice compared to the control groups.

Disialoganglioside (GD2) is a glycolipid antigen overexpressed on the surface of solid tumor cells, including neuroblastoma cells [55]. To improve targeting, silica nanoparticles were conjugated with an antibody targeting GD2 [19]. Following IV injection, the targeted delivery of the anti-GD2-NPs was confirmed by labelling NPs with FITC fluorescent dye. There was a 13.9-fold increase in the fluorescence intensity from tumors targeted with anti-GD2-NPs than control NPs with no antibody formulation. Following these positive results, the group investigated the therapeutic effects of miR-34a loaded anti-GD2-NPs in a neuroblastoma mouse model. After three systemic injections with the NP therapy or control NP, bioluminescent tumor monitoring and ex vivo tumor volume and weight measurements confirmed a significant reduction in tumor growth in mice treated with the anti-GD2–NPs loaded with miR-34a [19].

Silica nanoparticles were also used to deliver both miR-204-5p and oxaliplatin (OXL) combined to colon cancer [33]. MiR-204-5p is downregulated in colon cancer and targets many genes dysregulated in cancer, including RAB22A, HOXA10 and Bcl-2 [33]. OXL is a platinum-based chemotherapy agent commonly used in the treatment of colon cancer [56]. These nanoparticles were polyethyleneimine (PEI)-based mesoporous silica (MSN), conjugated with hyaluronic acid (HA). OXL was loaded in the MSN pores, while miR-204-5p was loaded via the PEI. The HA provided tumor-targeted delivery by binding to CD44 on colon cancer cells. The loaded nanoparticles were coined OXsi–HMSN. When either miR-204-5p or OXL were delivered without encapsulation, or individually encapsulated in vivo, neither therapy had a significant impact on tumor growth. However, OXmi–HMSN caused a significant inhibition of growth, with the induction of apoptosis confirmed ex vivo.

In normal tissue, tumor suppressor miR-100 facilitates the upregulation of p53 and caspase-3 cell apoptotic signalling through targeting mTOR [57]. Interestingly temozolomide (TMZ) works by activating the same apoptotic pathway. Alternatively, miR-21 is a powerful oncomiR that is overexpressed in GBM. Overexpression of miR-21 in cancer interrupts apoptotic signalling and dampens drug sensitivity by direct downregulation of phosphatase and tensin homolog (PTEN) and programmed cell death protein 4 (PDCD4) [58]. Co-delivery of both miR-100 and anti-miR-21 enhanced the effects of TMZ therapy when introduced into GBM cells [31]. To evaluate the therapeutic efficacy of this combined approach, a pre-clinical study was carried out using a murine GBM xenograft model. Due to the fragile nature of miRNA and anti-miRNA, they were encapsulated in gold–iron oxide nanoparticles (poly-GIONs). These nanoparticles were coated with β cyclodextrin–chitosan (CD–CS) hybrid polymer and with PEG-T7 peptide (T7-polyGIONs) [31]. These modifications allowed for sufficient loading of the miRNAs and for targeted delivery to GBM tumors. To avoid the BBB, the group investigated intranasal delivery of the therapeutic nanoparticles. Animals treated with T7-polyGIONs loaded with miR-100 and anti-miR-21 in combination with systemic TMZ therapy had improved survival compared to animals treated with one therapy alone or no therapy at all. The group also showed the feasibility of using intranasal delivery to circumvent the BBB and treat brain cancer.

MiR-655-3p is a TS miRNA that originates from the polycistronic miRNA gene cluster on human chromosome 14 [59]. Many of the miRNA derived from this chromosome cluster are downregulated in various cancers and have potent tumor suppressor capacities [60]. The anti-tumor activity of this therapeutic miRNA was investigated in liver metastases originating from primary colorectal tumors [32]. Nanoscale coordination polymers (NCPs) are hybrid nanoparticles consisting of metal ions and organic bridging ligands [61]. NCPs were loaded with a synthetic miR-655-3p mimic and the commercially available chemotherapy reagent, oxaliplatin. After IP injection, tumor growth was suppressed in the NCP–miR-655-3p/OXA group compared to the control groups. This was confirmed by ex vivo fluorescent imaging in which the tumor signal intensities were lower in the treatment group. This supports the rationale that delivering nanoparticles loaded with therapeutic miRNA and cytotoxic drugs provides a viable and potent treatment option for advanced cancer, in this instance, liver metastases.

## 5. Clinical Trials

Due to the infancy of the field, there have been only two clinical trials treating patients with liver cancer [62] and malignant pleural mesothelioma (MPM) [63] using tumor suppressor miRNAs encapsulated in nanoparticle formulations.

MiR-34a has repeatedly been studied as a potent tumor suppressor in a variety of cancers, including lung, brain and liver [19,20,24,25]. MiR-34a encapsulated in a liposomal formulation, coined MRX34, was tested as a viable TS miRNA delivery therapy in a preclinical liver cancer study. After IV injection of MRX34 in two different orthotopic liver tumor models, results confirmed high levels of miR-34a reached the liver cancer and the therapy inhibited tumor growth. MRX34 was also tested in an immunocompetent liver cancer model and did not stimulate the upregulation of key immune cytokines [35]. Based on this data, the company Mirna Therapeutics, Inc. carried out a first-in-human Phase I clinical trial to test the dose, safety and pharmacokinetics of MRX34 [62]. Cancer patients with previously untreatable solid tumors (majority with liver cancer) were enrolled in the study (n = 47). Due to adverse events when MRX34 was injected, a bi-weekly dosage schedule could not be maintained. Ultimately, after 5 cases of severe adverse immune-related effects, the trial had to be terminated [62]. This highlights the critical importance of developing a delivery vector that can evade the host immune system.

Bacterial minicells are a relatively new nanoparticle delivery system, with currently one pre-clinical study using bacterial minicells for delivery of a tumor suppressor miRNA. This has, however, progressed to clinical trial. MiR-16 is significantly downregulated in MPM compared to healthy controls [29]. In vitro experiments upregulating miR-16 in MPM cell lines demonstrated growth inhibition, suggesting miR-16 could have a therapeutic effect in vivo [29]. To test this, miR-16 was loaded into bacterial minicells and administered into a human xenograft model of MPM via IV injection. The bacterial minicells were modified with an EGFR antibody to provide tumor-targeting to the EGFR positive tumors. Tumor growth was inhibited after systemic administration of a low dose of the miR-16 minicells [29]. Based on this, a first-in-human Phase I dose-escalation study was carried out in MPM patients who had previous unsuccessful chemotherapy [63]. The miR-16-loaded minicells with EFGR targeting were coined “TargomiRs”. The main aims of this Phase I study were to establish the maximum tolerated dose, frequency of administration, duration of response and survival response. Twenty-six patients were given at least one TargomiR dose with dose-limiting toxicities observed, including anaphylaxis, infusion-related inflammation and cardiac events, e.g., cardiomyopathy. The maximum tolerated dose was 5 × 10^9^ TargomiRs per week. The mean overall survival was 200 days, with 5% of patients having a partial response, 68% of patients having stable disease and 27% of patients having progressive disease [63]. Due to the small patient number of this clinical study, the authors suggested confirmation of trial outcomes in a larger sample group and also coupling the therapy with chemotherapy or immune inhibitor drugs.

## 6. Discussion

Since the first discovery of these short non-coding RNAs less than 30 years ago, the scope and importance of miRNA have been building exponentially. It is clear that the NP delivery of TS miRNA to the tumor site presents a novel and attractive avenue for cancer therapy. This review highlights some of the key considerations in nanoparticle development to support ultimate clinical translation, including loading ability, stability in vivo, tumor targeting, low toxicity and immunogenicity.

The cancers that have been most extensively studied are brain malignancies, including glioblastoma and neuroblastoma [19,30], and lung cancers [21] (Table 1). Many tumors have the inherent ability to spread from the primary site to distant areas of the body, making them extremely difficult to treat. Encouragingly, the use of TS miRNA to treat cancer metastases has been tested in vivo in lung metastases of melanoma [20] and liver metastases of colorectal cancer [32].

Although most inorganic based NPs and bacterial minicells are not naturally tumor-specific, many methods have been employed to achieve the tumor-homing capability of these NPs, including conjugation with an antibody targeting the GD2 antigen overexpressed on neuroblastoma cells [19], conjugation with an antibody targeting the cancer-specific antigen EGFR [16], and conjugation with HA, which targets CD44 on colon cancer cells [33]. PAMAM dendrimers have also been encapsulated by NK–EVs as NK cells have innate tumor homing capacity [15]. The disadvantage of using inorganic nanoparticles is the tendency to non-specifically accumulate in healthy tissues, including the liver and kidneys. Interestingly Wu et al. [34], created an inorganic formulation coined BOMB that utilised environmental stimuli to fully degrade the nanoparticle after delivery at the tumor site.

Many studies using LNPs also revealed build up in major organs. This may be due to the reactivity of cationic lipids with anionic cell membrane proteins. In an effort to overcome this, one group employed a cationic lipid formulation with a neutral lipid coating [26]. Although off-target distribution was observed in major organs, including the lung, liver and spleen, toxicity studies suggested that the LNP was well tolerated. In contrast, Gokita et al. [37] showed AST levels were elevated in all mice receiving cationic LNPs compared to healthy control mice, suggesting toxicity related to the LNPs. In addition, the clinical trial using a liposomal formulation to deliver miR-34 to patients with previously untreatable solid tumors was terminated due to five cases of adverse immune-related effects [62].

Delivery to non-target cells and the potential for immunogenicity is a challenge in this field. There is increased interest in MSC-derived EVs due to the potential for innate tumor targeting and ability to bypass the host immune response based on the characteristics of the secreting cell. One promising study reported a significant reduction in tumor activity after systemic injection of MSC–EVs loaded with TS miR-379 [12]. The immunogenicity and toxicity of miRNA encapsulated EVs were tested in healthy immunocompetent mice (cancer-free) by Zhu et al. [64]. No toxicity or immune response was observed when mice were dosed with HEK293T-EVs loaded with miR-199a-3p via IV or IP injections over a 3-week time course. This supports the rationale that EVs could provide a less immunogenic option as a miRNA delivery vehicle. MSC- and immune cell-derived EVs are of major interest due to a potential capacity to bypass the host immune response, in combination with inherent tumor targeting.

Due to the relatively early stage of this field of research, many studies have employed transfer of one single miRNA, but for increased potency and translational potential, it is likely that a combinatorial approach will be more beneficial. The potential of this approach was demonstrated using a transcript containing three miRNAs, with synergistic tumor-suppressive capacity to increase the potency of the treatment [14]. Similarly, Chen et al. [20] combined a TS miRNA with multiple siRNAs for the treatment of lung metastases. A number of studies have also combined miRNA–NPs with standard of care chemotherapeutics, including doxorubicin [13] and oxaliplatin [32,33], to provide a synergistic treatment response. Developing on this rationale, Sukumar et al. [31] employed an NP formulation that combined three different aspects: a TS miRNA, an antisense miRNA to combat an oncomiR, and the chemotherapy agent TMZ to provide the most potent treatment for GBM. These advances in formulation capabilities, coupled with an increased understanding of factors controlling NP uptake and interaction with immune cells, will support the development of exciting novel therapeutic strategies to combat metastatic cancers with limited treatment options.

## Figures and Tables

**Table 1:** Pre-clinical studies utilising various nanoparticle formulations to deliver tumor suppressor miRNA. Intravenous (IV), intratumoral (IT), intraperitoneal (IP) retro-orbital (RO), doxorubicin (DOX), oxaliplatin (OXL), protein A disintegrin and metalloproteinase 15 (A15), human embryonic kidney 293 cells (HEK-293), liposome-polycation-hyaluronic acid (LPH), single chain antibody fragments (ScFv), nanoscale coordination polymers (NCPs), oral potentially malignant disorders (OMPD), exosome-mimetic nanosystems (EMNs), coated cationic lipid (CCL), NK cell–EVs with a biomimetic core shell (NN/NK–EV cocktail), biodegradable oncosensitive megamer based (BOMB), lipid nanoparticles (LPH), oral squamous cell cancer (OSCC).

Cancer Type	miRNA(s)	Delivery Vehicle	Route	Study Outcome	Ref.
Breast cancer	miR-379	MSC-EVs	IV	Significant reduction in tumor volume	[12]
	miR-159, DOX	Monocyte-EVs with A15	IV	Best therapeutic response after co-delivery of DOX and miR-159	[13]
	let-7a	HEK-293-EVs with GE11 peptide	IV	Tumor growth suppression	[23]
Lung cancer	miR-34a or let-7b	Neutral lipid emulsion	IV	Significantly lower tumor burden after treatment	[24]
	miR-133b	Cationic Lipoplexes	IV	Significant increase in premiR-133b expression at lungs	[21]
	miR-34a	Neutral lipid reagent	IT, IV	Large areas of tumor necrosis	[25]
	miR-660	CCL nanoparticles	IP, IV	Tumor growth significantly reduced	[26]
	miR-29b	Cationic lipoplexes	IV	Significantly smaller tumors after treatment	[27]
	miR-145	EMNs	IP, RO	After IP injection EMN signal detected at tumor site	[28]
	miR-16	Bacterial minicells	IV	Inhibition of tumor growth	[29]
Glioblastoma	miR-146b	MSC-EVs	IT	Significant decrease in tumor volume	[30]
	miR-124, -128, -137 (cluster 3)	Tumor derived EVs	IT	Significant increase in survival	[14]
	miR-100 and anti-miR-21	Gold-iron oxide nanoparticles	Intranasal	Progressive accumulation of NPs in the prefrontal cortex and longer survival	[31]
Neuroblastoma	miR-34a	Silica nanoparticles	IV	Significant reduction in tumor growth	[19]
	let-7a	NN/NK-EV cocktail	IV	Significantly lower tumor bioluminescence signal	[15]
Melanoma lung metastases	miR-34a and siRNAs	LPH nanoparticles with ScFv	IV	Co-delivery of miRNA and siRNA additively suppressed tumor growth	[20]
Colorectal liver metastases	miR-655-3p and OXL	NCPs	IP	Suppression of liver tumor development	[32]
Colorectal cancer	miR-204-5p and OXL	Silica nanoparticles	IV	Therapeutic agents combined had best therapeutic efficacy	[33]
Liver cancer	miR-122	BOMB nanoparticles	IV	Lower tumor volumes in treatment group	[34]
	miR-34a	Liposomal formulation	IV	Tumor growth inhibited	[35]
OPMD	miR-185	MSC-EVs	Topical	Reduced the incidence of transformation to OSCC	[36]
Pancreatic cancer	miR-634	LNPs	IV	Significant reduction in tumor growth	[37]
